# Point-of-Care Testing in Chronic Kidney Disease of Non-Traditional Origin: Considerations for Clinical, Epidemiological, and Health Surveillance Research and Practice

**DOI:** 10.5334/aogh.3884

**Published:** 2023-02-01

**Authors:** Miranda Dally, Juan José Amador, Jaime Butler-Dawson, Damaris Lopez-Pilarte, Alexandra Gero, Lyndsay Krisher, Alex Cruz, Daniel Pilloni, Joseph Kupferman, David J. Friedman, Benjamin R. Griffin, Lee S. Newman, Daniel R. Brooks

**Affiliations:** 1 Center for Health, Work, & Environment, Colorado School of Public Health, University of Colorado, Aurora, CO; 2 Department of Environmental and Occupational Health, Colorado School of Public Health, University of Colorado, Aurora, CO; 3 Department of Epidemiology, Boston University School of Public Health, Boston, MA; 4 Pantaleon, Guatemala City, Guatemala; 5 Division of Nephrology, Department of Medicine, Beth Israel Deaconess Medical Center, Harvard Medical School, Boston, MA; 6 Center for Vascular Biology Research, Beth Israel Deaconess Medical Center, Harvard Medical School, Boston, MA; 7 Division of Nephrology, University of Iowa, Iowa City, Iowa, USA; 8 Division of Pulmonary Sciences and Critical Care Medicine, Department of Medicine, School of Medicine, University of Colorado, Aurora, CO; 9 Department of Epidemiology, Colorado School of Public Health, University of Colorado, Aurora, CO

**Keywords:** Chronic kidney disease, point-of-care, health surveillance, epidemiology, clinical services

## Abstract

**Purpose:**

As the prevalence of chronic kidney disease of non-traditional origin (CKDnt) rises in low-resource settings, there is a need for reliable point-of-care creatinine testing. The purpose of this analysis was to assess the accuracy of two commonly used point-of-care creatinine devices, the i-STAT handheld (Abbott, Princeton, NJ, USA) and the StatSensor Creatinine (Nova Biomedical, Waltham, MA, USA) in comparison to venipuncture serum creatinine measures. The affordability, sensitivity, specificity, ease of use, and other considerations for each device are also presented.

**Methods:**

Three paired data sets were compared. We collected 213 paired i-STAT and venipuncture samples from a community study in Nicaragua in 2015–2016. We also collected 267 paired StatSensor Creatinine and venipuncture samples, including 158 from a community setting in Nicaragua in 2014–2015 and 109 from a Guatemala sugarcane worker cohort in 2017–2018. Pearson correlation coefficients, Bland-Altman plots, and no intercept linear regression models were used to assess agreement between point-of-care devices and blood samples.

**Results:**

The i-STAT performed the most accurately, overestimating creatinine by 0.07 mg/dL (95% CI: 0.02, 0.12) with no evidence of proportional bias. The StatSensor Creatinine performed well at low levels of creatinine (Mean (SD): 0.87 (0.19)). Due to proportional bias, the StatSensor Creatinine performed worse in the Nicaragua community setting where creatinine values ranged from 0.31 to 7.04 mg/dL.

**Discussion:**

Both devices provide acceptable sensitivity and specificity. Although adequate for routine surveillance, StatSensor Creatinine is less accurate as the values of measured creatinine increase, a consideration when using the point-of-care device for screening individuals at risk for CKDnt. Research, clinical, and screening objectives, cost, ease of use, and background prevalence of disease must all be carefully considered when selecting a point-of-care creatinine device.

**Conclusion:**

POC testing can be more accessible in resource-limited settings. The selection of the appropriate device will depend on the use-case.

## Introduction

As the global burden of disease has increased [1], so has the demand for diagnostic tests that are essential for identifying patients, determining prognosis, monitoring treatment, and assessing the efficacy of prevention [2]. Because traditional diagnostic laboratory tests are time-consuming and require complex infrastructure, skilled technicians, and a stable supply of electricity, they are not necessarily well-suited for meeting the increasing demands for timely diagnosis in low- and middle-income countries [3]. In response, point-of-care (POC) testing has emerged as a way to expedite diagnostic testing without the need for services of a remote clinical laboratory [4].

For similar reasons, POC devices have also been used in epidemiological studies of chronic disease prevalence and etiology. One such setting is in Central America in response to the epidemic of chronic kidney disease of non-traditional origin (CKDnt), which disproportionately affects young males who work in hot and humid climates and members of agricultural communities [5]. As the name suggests, causes of the disease are not well understood but are not explained by diabetes, hypertension, or other established factors [5,6]. To better understand the etiology, breadth, and clinical course of the disease, researchers have been increasingly conducting field studies [7] that assess serum creatinine values, a measure of renal function. Similarly, clinicians often examine patients’ creatinine levels in remote or isolated areas to make decisions about clinical treatment and disease management. Some agribusinesses have implemented pre-employment and mid-harvest creatinine screening of at-risk worker populations as awareness of CKDnt has grown [8]. Regardless of the purpose, a POC test may be the most feasible method of assessing kidney function in these resource-limited settings.

Creatinine POC devices have typically shown acceptable agreement with gold standard laboratory measurements when used in emergency departments, intensive care units, and other healthcare settings in the United States [9]. However, in field studies in Nicaragua [10] and Guatemala [11] researchers from Boston University and University of Colorado have independently found clinically meaningful discrepancies between POC-measured creatinine and lab values, in some cases leading to the development of a POC-device correction factor [11]. Understanding potential differences in accuracy and other characteristics of POC devices is of great importance when weighing the cost-benefits of using such a device.

In this paper, we assessed the accuracy, cost, and practical application of two POC creatinine devices commonly used for CKDnt research, the i-STAT handheld (Abbott, Princeton, NJ, USA) and the StatSensor Creatinine (Nova Biomedical, Waltham, MA, USA) in comparison to venipuncture creatinine measures assessed in certified labratories. We also sought to validate a previously published correction factor suggested for the StatSensor Creatinine [11]. We more broadly assessed the practicality of each device, examining benefits and limitations using the World Health Organization (WHO) ASSURED (Affordable, Sensitive, Specific, User-friendly, Rapid and robust, Equipment-free and Deliverable to end-users) criteria [12]. By presenting this information, we hope to provide information that will be valuable for those considering the use of POC devices for assessing creatinine values in low resource settings.

## Methods

### Point-of-care devices

We evaluated two commonly used POC devices that collect capillary blood and measure creatinine, the StatSensor Creatinine and the i-STAT handheld. Both devices use an enzymatic/amperometric method using whole blood obtained by fingerstick. The StatSensor Creatinine uses test strips, whereas the i-STAT handheld uses CHEM8+ cartridges.

### Data collection

Data for this analysis came from three separate studies conducted by different research teams. The first study examined kidney function in an occupationally based study among manual sugarcane cutters in Guatemala. The additional studies examined kidney function in two community populations in Nicaragua. Samples for all studies were collected by research personnel trained in the use of the devices following the manufacturers’ instructions.

Workers in the Guatemalan cohort cut sugarcane for a large agribusiness in Southwest Guatemala during the 6-month harvest season in 2017–2018. The agribusiness recruits workers from areas surrounding the sugar mill as well as migrant workers. The company has a hiring policy requiring sugarcane cutters to have a pre-employment test of creatinine in order to calculate their estimated glomerular filtration rate (eGFR). An eGFR greater than or equal to 90 mL/min per 1.73 m^2^ is required to determine job placement and employability. The workers included in this analysis were part of a study spanning the 2017–2018 harvest, in which two workgroups were randomly selected to participate. Participants were all males and 18 years of age or older. For one study workgroup, in November 2017, paired fingerstick StatSensor Creatinine and venipuncture measurements were collected prior to the start of the work shift to measure creatinine (n = 109). The 2017–2018 study was approved by the University of Colorado Multiple Institutional Review Board (COMIRB) and the ZUGUEME Comité de Ética Independiente in Guatemala.

The first Nicaragua community study consisted of residents of a town in the province of León who had originally participated in a case-control study in 2008 [13]. In 2015, venipuncture samples for laboratory analysis and fingerstick samples using the StatSensor Creatinine for POC analysis of creatinine were collected from 158 surviving participants with and without CKD. The second Nicaragua community study consisted of families in which multiple members had CKD and resided in the rural areas of a city in the province of Chinandega during 2014–15 [14]. For a family to enroll in the study, at least two first-degree relatives were required to have a serum creatinine level ≥ 1.5 mg/dL (males) or 1.4 mg/dL (females) at time of enrollment. Once a family was included, any additional family member aged 18 years or older could participate regardless of CKD status. Venipuncture samples for laboratory analysis and fingerstick samples using the i-STAT for POC analysis of creatinine were collected from 213 participants in 24 families. Both study protocols were approved by the institutional review boards at Boston University Medical Center and the Nicaraguan Ministry of Health.

The protocol for this secondary analysis of data was reviewed and approved by the Colorado Institutional Review Board.

### Laboratory methods

Detailed data collection and laboratory methods have been published in Kupferman, et al. 2016 (Nicaragua) [14] and Griffin, et al. 2018 (Guatemala) [11]. Briefly, during all studies, venipuncture blood samples were drawn concurrently with the whole blood capillary measures via fingerstick. The samples from Nicaragua were analyzed by the Centro Nacional de Diagnóstico y Referencia (CNDR) using the Cobas Integra 400 Plus (Roche Diagnostics International Ltd., Rotkreuz, Switzerland), which measures creatinine concentration by the compensated kinetic alkaline picrate method (IDMS standardized). The samples from Guatemala were analyzed by Herrera Llerandi Laboratory (HLL) using the Creatinine Jaffe Generation 2 method, which is also a kinetic alkaline picrate method.

### Statistical methods

Following convention, in our analysis, laboratory-analyzed samples were considered the gold standard despite being subject to error. To assess agreement between the POC devices and paired venipuncture samples, we first ran Pearson correlation coefficients. Paired t-tests were used to compare the mean POC and venipuncture creatinine measures, giving an estimate of the bias of the POC devices. Agreement was visualized using Bland-Altman plots [15]. The functional form of the relationship between POC and venipuncture samples was assessed using cubic smoothing splines with 4 degrees of freedom.

To validate the previously calculated StatSensor Creatinine correction factor presented by Griffin, et al. 2018 [11], we applied the published correction factor of 0.7775 to the Nicaragua StatSensor Creatinine data as well as the pre-shift Guatemala data. Adjusted mean POC creatinine measures were compared to the mean creatinine measures of the paired venipuncture samples. We then subsetted the analysis to only include creatinine values within the range of the Griffin analysis (creatinine < 2.59 mg/dL) in order to make the studies more comparable.

Additionally, using the methods employed by Griffen et al., 2018 [11], sample specific correction factors for POC creatinine from the i-STAT handheld and StatSensor Creatinine in Nicaragua and pre-shift StatSensor Creatinine in Guatemala were calculated by regressing venipuncture creatinine measures on POC estimates in a linear regression without intercept model. The resulting coefficients were compared to (1) provide comparisons between devices and (2) provide sample specific comparisons within devices.

All data analyses were performed using R version 3.4.3 [16].

### ASSURED criteria

The WHO has published criteria summarizing the ideal characteristics for a POC test in resource-limited settings [12]. Following the acronym ASSURED, the test must be affordable, sensitive (providing few false negatives), specific (few false positives), user-friendly (simple to perform needing minimum training), rapid and robust (does not require refrigeration for storage of samples), require no ancillary equipment, and delivered to those who need it. A full discussion of the ASSURED criteria is presented by Drain et al. [4] The ASSURED criteria were applied to the POC devices discussed in this paper.

## Results

On average, the creatinine levels in the Nicaragua studies measured higher than those in the Guatemala studies, likely due to study design and population differences, particularly the intentional inclusion of persons with elevated creatinine levels (Table 1).

**Table 1:** Mean and standard deviation of creatinine values from the point-of-care devices and laboratory analysis used in the Nicaraguan and Guatemalan studies. Average differences and 95% confidence intervals between laboratory values and unadjusted point-of-care devices.


	STATSENSOR (GT) N = 109		STATSENSOR (GT) N = 192		STATSENSOR (Nic) N = 158		I-STAT (NIC) N = 213	

POPULATION	SUGARCANE CUTTERS, PRE-WORK SHIFT		SUGARCANE CUTTERS, POST-WORK SHIFT^1^		COMMUNITY POPULATION, CASE-CONTROL STUDY FOLLOW-UP		COMMUNITY POPULATION, HIGH-RISK FAMILIES^2^	
			
	MEAN (SD OR *95% CI*)	P-VALUE	MEAN (SD OR *95% CI*)	P-VALUE	MEAN (SD OR *95% CI*)	P-VALUE	MEAN (SD OR *95% CI*)	P-VALUE

Lab creatinine measure	0.87 (0.19)		0.88 (0.21)		1.10 (1.04)		1.89 (1.98)	
			
Unadjusted POC creatinine measure	0.85 (0.22)		1.08 (0.35)		1.58 (1.60)		1.96 (2.20)	

*Difference (Serum lab – POC unadjusted)*	*0.02 (–0.01, 0.05)*	*0.14*	*–0.20 (–0.24, –0.17)*	*<0.001*	*–0.48 (–0.58, –0.38)*	*<0.001*	*–0.07 (–0.12, –0.02)*	*0.01*


^1^ Griffin, B.R., et al., Unadjusted point of care creatinine results overestimate acute kidney injury incidence during field testing in Guatemala. PloS one, 2018. 13(9): p. e0204614–e0204614. ^2^ Kupferman, J., et al., Characterization of Mesoamerican Nephropathy in a Kidney Failure Hotspot in Nicaragua. American Journal of Kidney Diseases, 2016. 68(5): p. 716–725.

### i-STAT handheld comparisons

**Figure 1:**
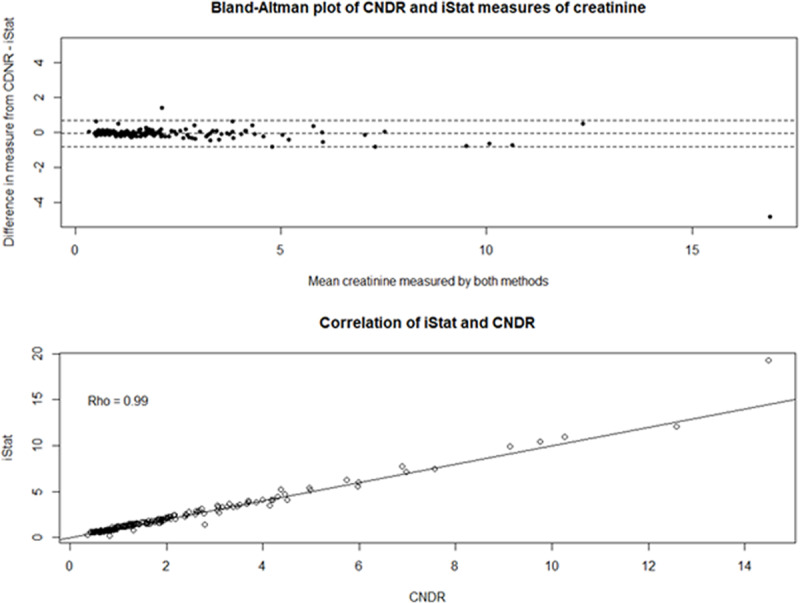
Top: Correlation and agreement between i-STAT point-of-care creatinine values and CNDR laboratory creatinine values from the 2015–2016 Nicaragua study. Bottom: Bland-Altman plot of agreement between i-STAT point-of-care creatinine values and CNDR laboratory creatinine values.

The 213 paired i-STAT and venipuncture samples exhibited excellent correlation between the two measurement techniques (ρ = 0.99) as well as good agreement (Figure 1, Top). On average, the i-STAT overestimated creatinine by 0.07 mg/dL (95% CI: 0.02, 0.12) compared to the lab measures. Examination of the Bland-Altman plot showed no evidence of proportional bias, indicating that the level of disagreement was consistent at all values of creatinine measured (Figure 1, Bottom).

### StatSensor Creatinine comparisons

**Figure 2:**
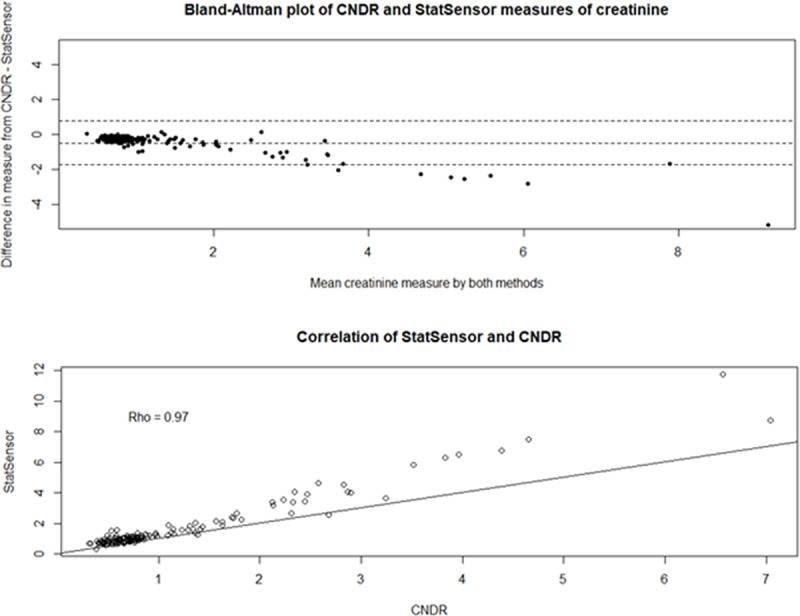
Top: Correlation and agreement between StatSensor Creatinine point-of-care creatinine values and CNDR laboratory creatinine values from the 2015 Nicaragua study. Bottom: Bland-Altman plot of agreement between StatSensor Creatinine point-of-care creatinine values and CNDR laboratory creatinine values.

There were 158 paired StatSensor Creatinine and venipuncture samples from the 2015 Nicaragua study. We observed excellent correlation between the measurements (ρ = 0.97), however there was obvious deviation from the line of agreement (Figure 2, Top). The Bland-Altman plot demonstrates proportional bias between measures taken on the StatSensor Creatinine and the venipuncture measures analyzed in the lab, indicating that the level of disagreement increases at higher creatinine levels. On average, the StatSensor Creatinine overestimated creatinine levels by 0.48 mg/dL (95% CI: 0.38, 0.58). When the StatSensor Creatinine values from the Nicaragua studies were outside of the range of those in the Griffin et al. 2018 Guatemala study, were removed (>2.59 mg/dL, n = 21), the resulting difference between venipuncture and POC measurement was reduced to 0.30 mg/dL (95% CI: 0.26, 0.33). This is in line with what was observed in the post-shift measures in the Guatemala 2017–2018 study which showed the StatSensor Creatinine overestimated by 0.20 mg/dL (95%CI: 0.17, 0.24) [11].

**Figure 3:**
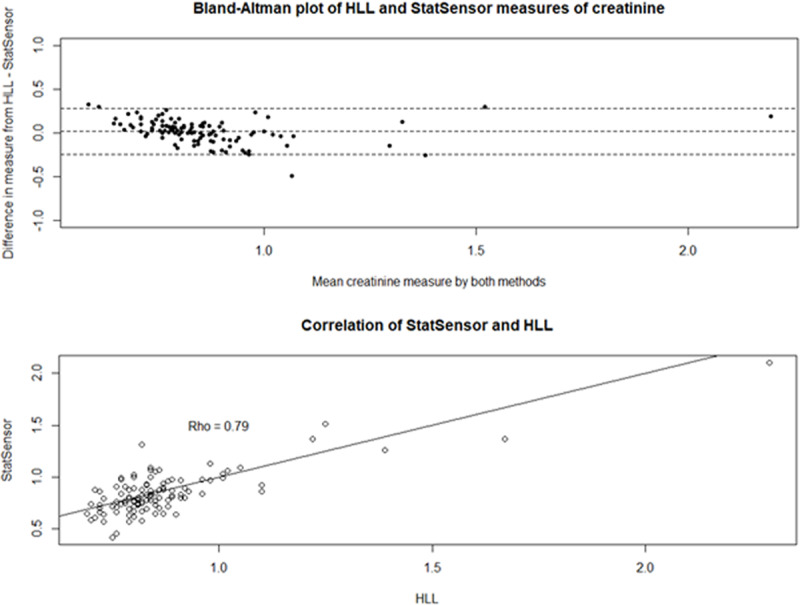
Top: Correlation and agreement between StatSensor Creatinine point-of-care creatinine values and HLL laboratory creatinine values from the pre-shift November 2017 Guatemala data. Bottom: Bland-Altman plot of agreement between StatSensor Creatinine point-of-care creatinine values and HLL laboratory cretainine values.

There were 109 paired pre-shift samples from the November 2017 Guatemala study. The correlation was good (ρ = 0.79) between the StatSensor Creatinine and the venipuncture values (Figure 3, Top). The Bland-Altman plot demonstrates no evidence of proportional bias (Figure 3, Top). On average the StatSensor Creatinine underestimated creatinine by 0.02 mg/dL (95% CI: –0.01, 0.05).

### Calculated correction factors

Correction factors were calculated for each of the three datasets. For the pre-shift data using the StatSensor Creatinine in Guatemala, the estimated correction factor was 1.00 (implying no correction). The correction factor for the StatSensor Creatinine Nicaragua data was 0.66 and for the subset StatSensor Creatinine Nicaragua data (creatinine < 2.59 mg/dL), the correction factor was 0.73. The estimated i-STAT correction factor was 0.92. A summary of these results can be found in Table 2.

**Table 2:** Correction factor for each of the studies based on linear regression with no intercept. Calculated adjusted point-of-care creatinine measurements based on the study-specific correction factor as well as application of the Griffin et al., 2018 correction factor. Mean and 95% confidence intervals for differences between adjusted point-of-care creatinine and laboratory creatinine values.

	
	LAB VALUE	POC VALUE	CORRECTION FACTOR LINEAR REGRESSION	CORRECTION FACTORGRIFFIN ET AL., 2018	ADJUSTED POCLINEAR REGRESSION	ADJUSTED POCGRIFFIN ET AL., 2018	DIFFERENCE LAB ANDADJUSTED POCLINEAR REGRESSION	DIFFERENCE LAB ANDADJUSTED POC GRIFFIN ET AL., 2018

StatSensor Creatinine November 2017 GuatemalaN = 109	0.87 (0.19)	0.85 (0.22)	1.00	0.78	0.85 (0.22)	0.66 (0.17)	0.02 (–0.01, 0.05)	0.21 (0.18, 0.23)
	
StatSensor Creatinine 2015 NicaraguaN = 158	1.10 (1.04)	1.58 (1.60)	0.66		1.04 (1.06)	1.23 (1.25)	0.06 (0.02, 0.09)	–0.14 (–0.19, –0.08)
	
StatSensor Creatinine 2015 Nicaragua – Subset^1^N = 137	0.77 (0.35)	1.06 (0.40)	0.73		0.78 (0.29)	0.83 (0.31)	–0.01 (–0.04, 0.02)	–0.06 (–0.09, –0.03)

i-STAT 2015-2016 NicaraguaN = 213	1.89 (1.98)	1.96 (2.20)	0.92		1.80 (2.02)		0.09 (0.05, 0.13)	


^a^ Assessing a creatinine cutoff of 1.5 mg/dL.

### Application of ASSURED Criteria

A summary of the ASSURED criteria applied to each POC device is shown in Table 3.

**Table 3:** Summary of WHO ASSURED criteria as applied to the i-STAT, StatSensor Creatinine, and laboratory measurements of creatinine. Pooled StatSensor Creatinine data was used to calculate sensitivity and specificity, with laboratory measurements as the gold standard.


CHARACTERISTIC	I-STAT	STATSENSOR CREATININE	LABORATORY

Affordable	$14,000 per device$20 per test for Cr and other serum measures	$3,600 per device$6 per Cr test	Local costs vary

Sensitive^a^	99%	100%	Gold standard with adequate laboratory infrastructure

Specific^a^	86%	92%	Gold standard with adequate laboratory infrastructure

User-friendly	✔	✔	Lab technician required

Rapid	2 minutes	30 seconds	Days to weeks

Equipment-free	Cooler in hot climate	Cooler in hot climate	Electricity and refrigeration

Deliverable	✔	✔	✖


^1^ Creatinine measures > 2.59 mg/dL (n = 21) were removed in the subset.

#### Affordability

As of 2021, the i-STAT, including the software and other necessary equipment, costs upwards of $14,000USD. Each cartridge costs about $20USD per sample when purchased in Central America, and provides results for creatinine, sodium, potassium, chloride, blood urea nitrogen, glucose, ionized calcium, carbon dioxide (TCO2), hematocrit, and hemoglobin. The StatSensor Creatinine costs approximately $3,600USD for the standard device package, plus $300USD per box of 50 creatinine test strips, or $6USD per test. Of note, this pricing is based on purchasing within the United States, in our case as part of a contract with an academic institution. Pricing may vary considerably. Both Abbott (iSTAT) and Nova Biomedical (StatSensor Creatinine) have product distributors in Latin America and other parts of the world in addition to the US and Europe, although costs for shipping and importation may be important considerations, especially if the distributor is not located in the same country.

#### Sensitivity

Sensitivity is a measure of a test’s ability to correctly identify true positives. We calculated the sensitivity of the POC devices for detecting creatinine levels of 1.5 mg/dL or above by comparing to laboratory measures (Table 4). For the i-STAT, sensitivity was calculated at 99%. The pooled StatSensor Creatinine data showed sensitivity of 97%. Both POC devices perform very well at identifying individuals whose true creatinine value is 1.5 mg/dL or above, as would be expected since both devices provided results that were slightly higher on average compared to laboratory values.

**Table 4:** Sensitivity, specificity, and positive predictive value of creatinine levels detected at a threshold of 1.5 mg/dL for StatSensor and i-STAT compared to laboratory measurement. StatSensor Creatinine—Sensitivity: 97%; Specificity: 95%; Positive predictive value: 71%. i-STAT—Sensitivity: 99%; Specificity: 92%; Positive predictive value: 90%.


≥1.5 MD/DL		LAB IDENTIFIED	

		< 1.5 MG/DL	

StatSensor Creatinine Identified	≥1.5 md/dL	29	12

	<1.5 mg/dL	1	225

i-STAT Identified	≥1.5 md/dL	86	10

	<1.5 mg/dL	1	116


#### Specificity

Specificity is a measure of a test’s ability to correctly identify true negatives. Using a threshold of 1.5 mg/dL, specificity was 92% for the i-STAT and 95% for the pooled StatSensor Creatinine. These values indicate that a few individuals who were below 1.5 mg/dL were falsely identified as having elevated creatinine when using the POC devices. Given the differences in distribution of creatinine levels between the populations in which the two devices were used (Table 5), a comparison of the difference in specificity between the devices is not appropriate. Specificity measurements are provided to demonstrate that both devices provide sufficient specificity.

**Table 5:** Distribution of measured creatinine values in each of the study populations.


	N	MEASURED CREATININE (MG/DL)			

		<1.3	1.3–1.49	1.5–1.7	>1.7

i-STAT (Nicaragua)	213	104 (48.8%)	13 (6.1%)	23 (10.8%)	73 (34.3%)

StatSensor Creatinine (Combined)	267	217 (81.3%)	9 (3.4%)	8 (3.0%)	33 (12.4%)

StatSensor Creatinine (Nicaragua)	158	113 (71.5%)	6 (3.8%)	7 (4.4%)	32 (20.3%)

StatSensor Creatinine (Guatemala)	109	104 (95.4%)	3 (2.8%)	1 (0.9%)	1 (0.9%)


The positive predictive value (PPV) of the test, the proportion of people who test positive who are truly positive for the condition, is an additional significant measure of test performance not included in the ASSURED criteria. The PPV was 90% for the i-STAT and 71% for the StatSensor Creatinine. These figures cannot be directly compared because the PPV is impacted by the prevalence of the condition in the tested populations, which differed significantly in these studies. The prevalence of a creatinine level of ≥1.5 mg/dL was 45% in the population in which the i-STAT was used compared to 15% for the population tested with the StatSensor Creatinine. Regardless, both devices provide sufficient PPV.

#### User-friendly

Both POC devices can be utilized by any operator trained on device use, including those without medical expertise. For example, with routine training, both practicing clinicians as well as research support staff with no clinical background in Guatemala and Nicaragua were able to use the StatSensor Creatinine and i-STAT successfully in the field. Neither the i-STAT nor StatSensor Creatinine require venipuncture, reducing the need for phlebotomists to perform the blood draw. However, in some settings the volume of blood required by the i-STAT may be prohibitive for fingerstick, such as workers with heavily calloused hands, in which venipuncture would still be required. If venipuncture is necessary for other tests or for storage of samples, the i-STAT can use blood from the venipuncture, obviating the need for a separate fingerstick [17].

It is not uncommon in regions where CKDnT is prevalent for temperatures to exceed 40°C, with humidity exceeding 90%. Both POC devices should be kept cool to avoid overheating the machinery in tropical conditions. It is common practice to keep the devices in a cooler when not in use in the field. The i-STAT can be operated at 16°C to 37°C and automatically shuts off when it senses temperatures beyond this range. in order to avoid inaccurate test values. The team in Nicaragua addressed this situation using several methods, including collecting samples in the morning before temperatures rose, carrying the device in a cooler, or keeping the device in an air-conditioned vehicle and collecting the sample with the participant adjacent. The StatSensor Creatinine can be operated from 15°C to 40°C; it will still function at high temperatures, though there may be a decrease in accuracy in the direction of higher creatinine levels. Both devices function appropriately in humidity ranges from 10% to 90%.

#### Rapid and robust

One of the biggest concerns about conventional laboratory-based testing is the long turn-around times and delays in returning test results to individuals [18]. Rapid turn-around time is defined as a process that is completed “in the same clinical encounter,” such that results can be provided back to the clinician, patient, or research subject instantly without loss to follow-up [3]. The StatSensor Creatinine can read creatinine results in 30 seconds. i-STAT results are available within minutes. Both POC devices are considered robust under the ASSURED criteria as the samples do not need to be prepared, centrifuged, and kept at appropriate temperatures for transport and storage, a common problem in rural or resource-limited areas.

#### No ancillary equipment

The StatSensor Creatinine runs on a rechargeable battery with a battery life of 8 hours while in use and the sealed creatinine reagent strips are stable for 12 months at 4–8°C and for three months at room temperature. The StatSensor Creatinine can run a minimum of 600 tests per replaceable battery life. Additionally, the device can store up to 400 tests before the data need to be downloaded. Data can be transferred via USB to a Microsoft Excel-based transfer software.

The Chem8+ cartridges used with the i-STAT are guaranteed stable for 60 days, up to 6 months once shipped from the warehouse, if kept refrigerated (2°C to 8°C); the shelf life of the cartridges decreases if stored at room temperature. This may limit the amount of viable time that the tests can be utilized, especially if they are being transported internationally. The i-STAT runs on a rechargeable battery and can store up to 1,000 test results. Disposable batteries can be used in the i-STAT while the rechargeable battery is charging. The fully charged battery will self-discharge in approximately 3 months. Data from the i-STAT can be transferred from either a USB connection or network connection.

#### Deliverable to those who need it

In resource-limited settings it is not always feasible for individuals to have their creatinine checked or monitored by accredited laboratories. The published use of StatSensor Creatinine and i-STAT POC devices shows their ability to be accessed by those who need it. Rapid readings allow for immediate action for patients with adverse clinical results. In fact, the experience in community settings in Nicaragua has been that the use of a POC device is a motivation to participate because many residents want to know their creatinine level but cannot otherwise obtain that information. In addition, participants in both countries prefer the POC device to the blood draw because of the much lower volume of blood required; this is especially true for workers prior to working their shift and for participants with CKD who frequently have anemia.

## Discussion

In this paper, we assessed the accuracy, affordability, and accessibility of two POC creatinine devices that have been used for CKDnT research. The use of POC devices can address challenges found in settings that are resource-constrained due to limited medical personnel, inadequate laboratory infrastructure, and inconsistent supply chains. While the i-STAT POC device provided the most accurate results, each POC had its advantages when used in a low-resource setting, consistent with what has been found in the clinical setting [19].

With increasing prevalence of CKD and the emergence of CKDnT in limited resource settings [20], there is a need for diagnostic tools to identify and monitor patients, as well as conduct epidemiological studies [2]. Unfortunately, the traditional tools used are not easily accessible in limited resouce settings [21]. Creatinine POC devices offer a more accessible alternative to traditional laboratory methods. Furthermore, in communities greatly affected by CKDnT, use of a POC device that allows immediate feedback on creatinine levels is an important motivation for enrollment and continued participation in epidemiological studies.

While the feasibility of their use have been demonstrated in a variety of settings, from hospital clinics [21], community settings [14], and workplaces [8], little guidance has been provided on which POC device is most appropriate to use. The answer to this question will depend on the desired outcome. In situations where a large number of individuals are being screened for CKDnT, such as for surveillance purposes or in occupational settings, the cost of the i-STAT may be prohibitive, while the StatSensor Creatinine would provide satisfactory results. However, in clinical or epidemiological settings where staging CKDnT or monitoring disease progression is of primary concern, the accurate measurement of the i-STAT would more likely be worth the cost. In epidemiological studies where misclassification of disease status may be a concern, selecting the more sensitive and specific device for the disease status of the population may be more appropriate than considering average differences in accurate point measurement. An additional consideration is whether other clinical tests are desired. The i-STAT cartridge that measures creatinine also returns results on a variety of other measures, with additional cartridges available for testing hematology, cardiac markers, and other blood parameters. Regardless, both machines are adequate alternatives to clinical laboratory measurements when clinical laboratories are difficult to access or lead to a delay in conveying data back to patients/participants.

The StatSensor Creatinine was originally designed to err high to avoid false negatives. While this is sufficient for categorization of individuals with CKDnT, it is necessary to consider the implications of a false positive. If workplaces are utilizing the StatSensor Creatinine to make hiring or job placement decisions, workers who are identified as borderline based on StatSensor Creatinine measures should be followed up with more accurate testing. While evidence presented here suggest that the StatSensor Creatinine should not be used if quantitative measures of creatinine are desired, if it will be used in this manner correction factors should be considered. As demonstrated in the analysis above, correction factors differ based on the range of creatinine values being measured. This presents difficulty in validating correction factors as they are sample dependent. We showed that the correction factor presented in Griffin et al. 2018 [11] was only valid when we limited the Nicaragua cohort to the creatinine values used to calculate the original correction factor. We suggest that if circumstances allow, any time a POC device is deployed, a small subset of concurrent samples should be run in the laboratory to understand potential biases. At the same time, the quality of the laboratory as a gold standard against which the POC devices are to be compared should also be considered. Not all labs in resource-poor settings will have the capacity to employ the IDMS-standardized method for analysis of creatinine. Even if the machinery is available, training and maintenance can also affect measurement accuracy.

Another consideration for the observed differences among the POC and venipuncture measurements could be due to differences between capillary and venous measurements of creatinine. Capillary measurements are more sensitive to changes in hydration status which is a possible explanation for why pre-shift measurements in Guatemala showed good agreement, while a correction factor is needed for post-shift measurements when workers experience higher degrees of dehydration. Another possibility to explain the difference is that hotter temperatures experienced in the afternoon are affecting the accuracy of the StatSensor Creatinine; although in one study, temperature effects did not seem to affect the accuracy of the i-STAT within the temperature ranges it is designed to operate [17]. Additionally, as noted above, laboratory results themselves may be subject to error and it can be difficult to disentangle what proportion of the variability between laboratory and POC measures should be assigned to the POC devices.

The primary limitation to this study is that it was based on a secondary evaluation of data collected for various other purposes in two separate populations, which limited our ability to make direct comparisons in accuracy between the i-STAT and StatSensor Creatinine. The preferred design would have been to collect samples for laboratory analyses and both POC devices from the same individuals. One consequence is that sensitivity, specificity, and positive predictive value measures cannot be directly compared between the POC devices because all these measures are affected by the distribution of creatinine levels, which differed in the study populations. In particular, as illustrated in Table 5, the StatSensor Creatinine had a substantially lower percentage of values than the iSTAT in the two categories that were closest to the threshold of 1.5 mg/dL, and was therefore more likely to be subject to misclassification (6.4% and 16.9%, respectively).

Additionally, conditions such as weather, time of collection, or other unidentified factors that may have affected the results likely differed between the studies. Finally, the gold standard venipuncture serum creatinine tests were conducted at two different laboratories using two different, albeit approved, clinical laboratory methods. Therefore, the results related to accuracy are best interpreted as an independent comparison of each POC device to the corresponding laboratory value. Other factors, such as cost, ease of use, and range of blood parameters that can be analyzed can appropriately be directly compared.
